# CD8 Positive T Lymphocyte Infiltration of Liver Metastases of Uveal Melanoma: A Case Report

**DOI:** 10.3389/fonc.2021.672660

**Published:** 2021-06-02

**Authors:** Naoki Takahashi, Kazuto Tajiri, Ko Kagoyana, Shinichi Tanaka, Ichiro Yasuda

**Affiliations:** ^1^ The Third Department of Internal Medicine, Faculty of Medicine, University of Toyama, Toyama, Japan; ^2^ Department of Dermatology, Faculty of Medicine, University of Toyama, Toyama, Japan; ^3^ Department of Diagnostic Pathology, Faculty of Medicine, University of Toyama, Toyama, Japan

**Keywords:** malignant melanoma, uveal melanoma, liver metastasis, CD8 positive T lymphocyte, host immune response

## Abstract

A 78-year-old Japanese man was referred for examination of multiple small nodules on his liver detected by magnetic resonance imaging (MRI). These small nodules were hyperintense on T1-weighted MRI, and were in hepatobiliary phase on gadolinium-ethoxybenzyl-diethylenetriamine pentaacetic acid enhanced MRI. Five years earlier, he had undergone curative enucleation of his left eye for uveal melanoma. US-guided biopsy revealed that the liver nodules were metastases of melanoma. Pathological examination also showed infiltration of CD8 positive T lymphocytes. The metastatic nodules remained unchanged for more than 2 years and he was not further treated. This pathology and clinical course suggest that the systemic immune response of the host could suppress hepatic metastases of uveal melanoma.

## Introduction

Malignant melanoma is a neoplasm derived from melanocytes. The most common primary site of this neoplasm is the skin, followed by the mucosa and eyes. Malignant melanoma is relatively rare in Asia and the prognosis of patients with metastatic melanoma is extremely poor. Uveal melanoma is a rare disease, with a reported annual incidence of 5.5-6 per million among Whites and 0.2-0.3 per million among Asians (1). Uveal melanoma is aggressive and metastasizes hematogenously, most frequently to the liver (60–87%), but also to the lungs (24–26%), skin (10 – 17%), bones (8.4–29%), and brain (4.2%) (2–4). Liver metastasis is the leading cause of death in patients with malignant melanoma, with early detection of liver metastases important in prolonging survival (5). Regarding with the development of liver metastasis from uveal melanoma, a study with autopsy cases suggested that hepatic micrometastasis can occur in advance clinical diagnosis of the primary tumor (6). The metastatic tumor develops with from infiltrative pattern to nodular growth pattern, and the infiltrating immune cells change from natural killer (NK) cells to CD3-positive lymphocytes according to tumor growth (7). Thus not only adoptive but also innate immune cells including NK cells contribute to the formation of tumor microenvironment (8). However, on the other hand, uveal melanoma is refractory to immunotherapy, the standard treatment for metastatic melanoma, with the median survival of patients diagnosed with liver metastases of uveal melanoma being only several months (9–11). A recent study reported that CD8 positive T lymphocytes expressing lymphocyte activation gene-3, rather than programmed death-1 or cytotoxic T-lymphocyte antigen 4, are involved in immune-checkpoint mechanism in uveal melanoma (12). Further investigation is required to improve the prognosis of uveal melanoma.

We encountered a patient with small liver metastases of uveal melanoma detected 5 years after surgical resection of the primary lesion. Despite a lack of treatment, these small liver metastatic nodules showed no evidence of progression for more than 2 years. Biopsies of the liver nodules showed infiltration of CD8 positive lymphocytes, suggesting that host immunity could suppress the progression of disease, depending on the status of liver metastases.

## Case Report

**Figure 1:**
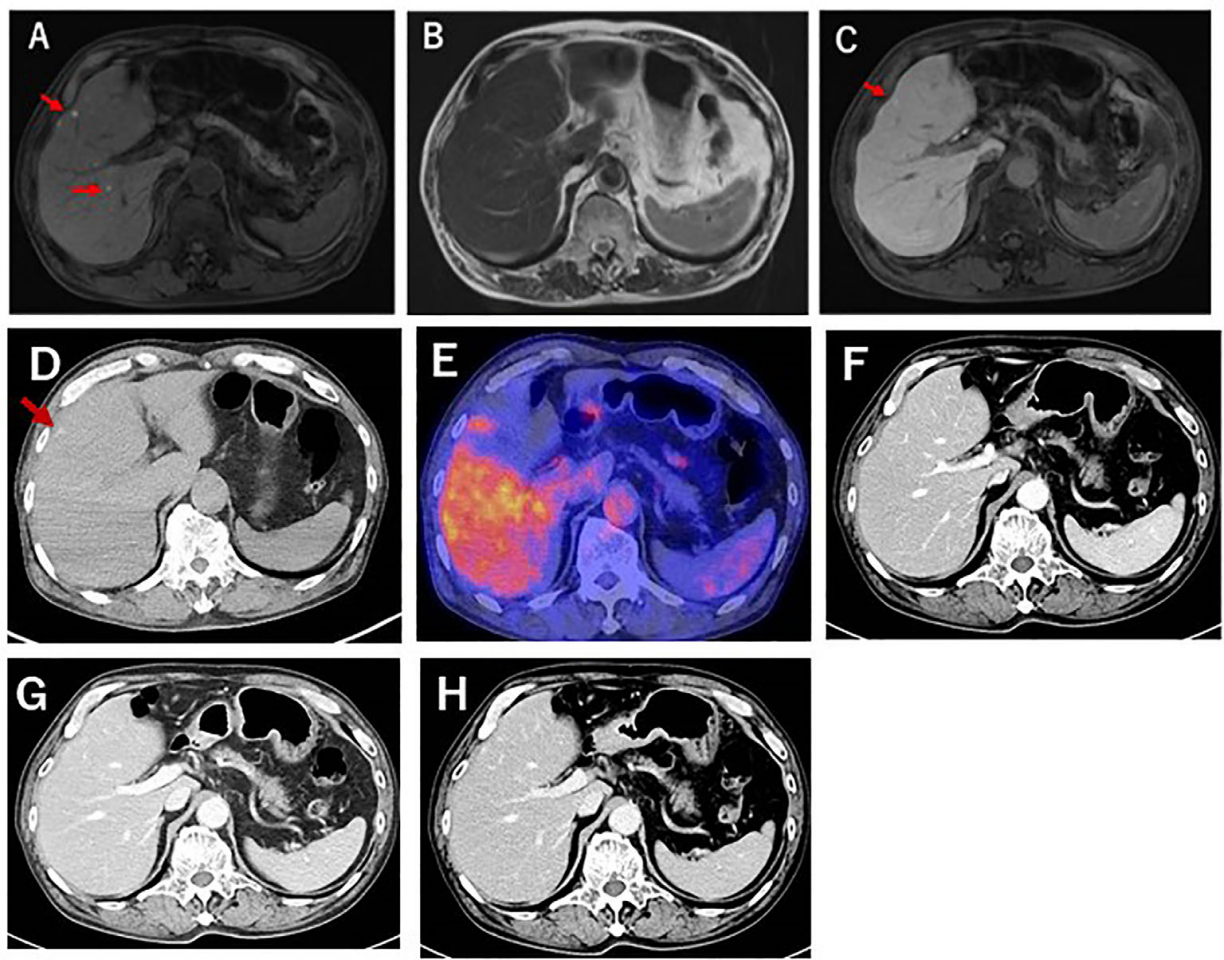
Gd-EOB-DTPA enhanced MRI of this patient. **(A)** T1-weighted MRI showing that the liver nodules (red arrows) were highly intense. **(B)** T2-weighted MRI, showing that the liver nodules were iso-intense. **(C)** Gd-EOB-DTPA-enhanced MRI, showing the showed were highly intense during the hepatobiliary phase. **(D)** Plain-CT examination 1 year before liver biopsy, showing small, high-density nodules. **(E)** FDG-PET CT examination at liver biopsy. No abnormal FDG uptake was detected. **(F–H)** Dynamic CT examination during the **(F)** arterial, **(G)** portal phase, and **(H)** equivalent phases. No enhancement was detected.

**Figure 2:**
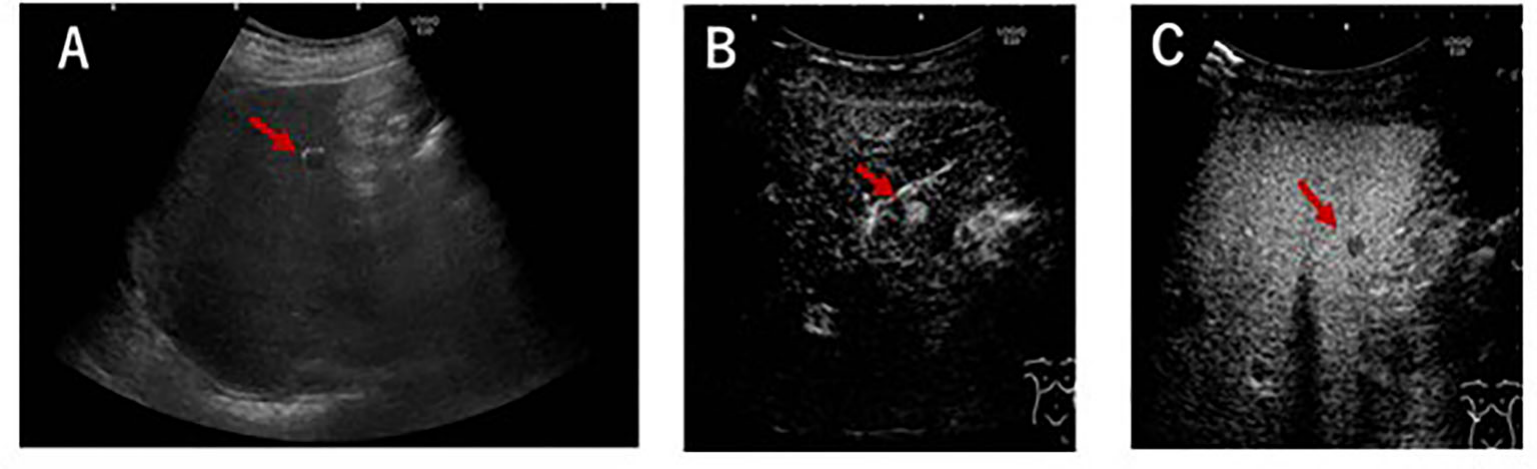
US findings. **(A)** B-mode US, showing tiny hypoechoic nodules. **(B, C)** Sonazoid enhanced US during **(B)** early and **(C)** delayed vascular phases. A perfusion defect was also observed during the delayed phase.

**Figure 3:**
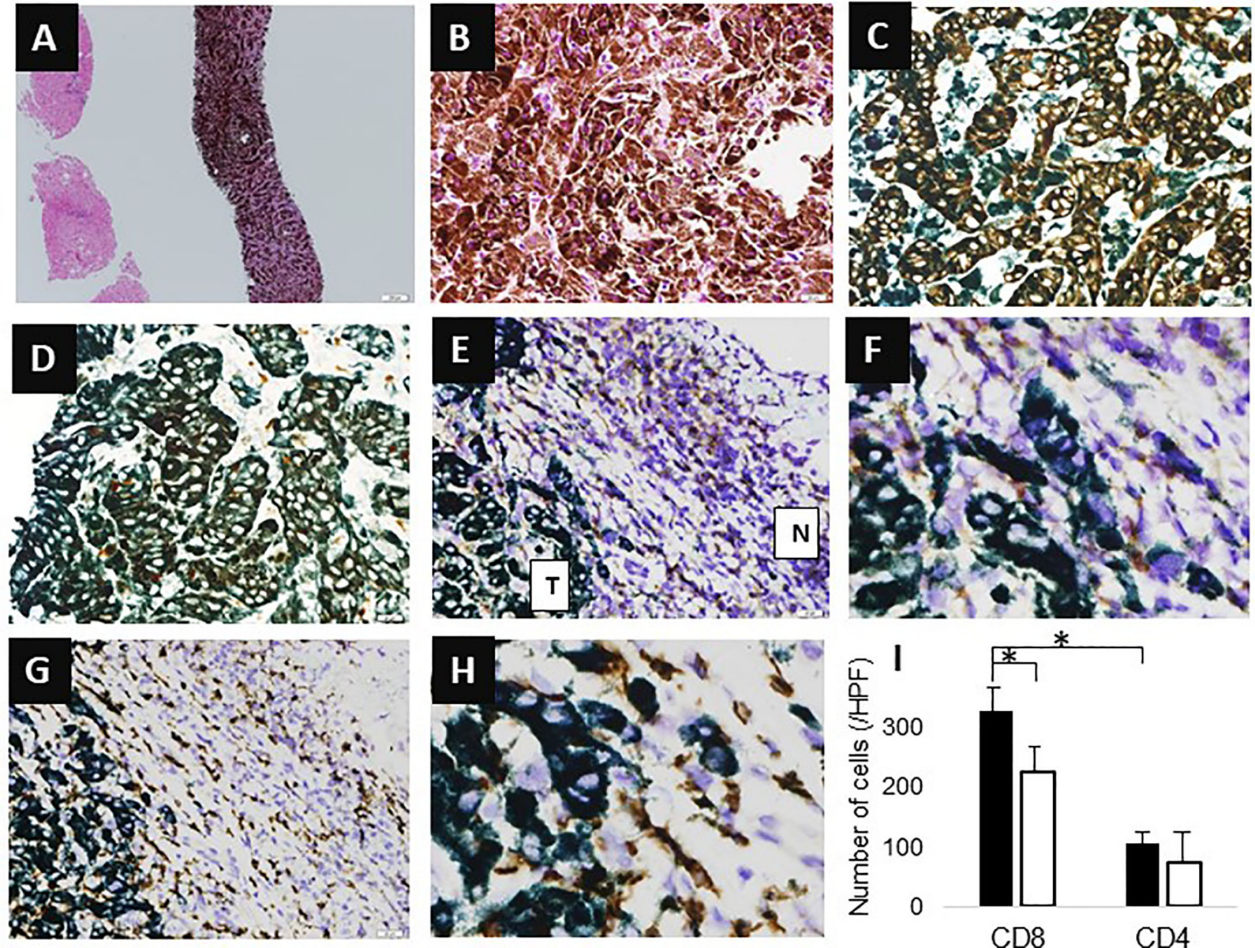
Histological findings. **(A, B)** Hematoxylin-eosin (HE) staining (**A**, X4; **B**, X200). **(C)** Melan-A staining (X400). **(D)** MIB-1 staining (X400). **(E, F)** CD4 staining (**E**, X100; **F**, X400); T indicates tumor area, N indicates non-tumor area. **(G, H)** CD8 staining (**G**, X100; **H**, X400). **(I)** Number of infiltrating cells. Y-axis represents the number of infiltrating cells. The average number of cells in three high power fields is shown. Black bar means the number of cells in tumor area, whereas white bar means that of non-tumor area. Asterisk indicates statistical significance (p<0.05).

A 78-year-old Japanese man was referred to our department for examination of multiple small nodules, of diameter 4-5 mm, detected by magnetic resonance imaging (MRI). These small nodules showed high-intensity on T1 weighted MRI (**Figure 1A**), iso-intensity on T2 weighted imaging (**Figure 1B**), and high-intensity on hepatobiliary phase of gadolinium-ethoxybenzyl-diethyleneaminepantaacetic acid (Gd-EOB-DTPA)-enhanced MRI (**Figure 1C**). Five years earlier, he had undergone curative enucleation of his left eye for uveal melanoma, but he was asymptomatic during this 5-year period. Laboratory data showed slight increases in the concentrations of hepatobiliary enzymes, including aspartate aminotransferase (32 U/L), alanine aminotransferase (26 U/L), alkaline phosphatase (383 U/L), γ−glutamyl transpeptidase (227 U/L), and total bilirubin (1.0 mg/dL). Serologic examination showed no evidence of hepatitis B or C virus infection or autoimmune liver injury. Annual evaluation by fluorodeoxyglucose positron emission tomography (FDG-PET)/computed tomography (CT) showed no evidence of abnormal FDG uptake during this 5-year period. Retrospective examination, however, identified small hyperdense small nodules on the plain phase of FDG-PET/CT at least 1 year earlier (**Figures 1D, E**). These nodules were not enhanced on dynamic CT (**Figures 1F–H**) but were hypoechoic on B-mode ultrasonography (US) (**Figure 2A**). On Sonazoid-enhanced US, these nodules showed strong enhancement during early arterial phase (**Figure 2B**) and were hypoechoic on delayed phase (**Figure 2C**), suggesting that these nodules were liver metastases of uveal melanoma.Macroscopic examination of US-guided biopsy specimens of the liver nodules showed black pigmentation (**Figure 3A**). Pathological evaluation showed proliferation of abnormally pigmented tumor cells (**Figure 3B**), which were positive for Melan-A (**Figure 3C**), HMB45 and S100 (data not shown), confirming that these nodules were hepatic metastases of uveal melanoma. The highly proliferative nature and aggressive phenotype of these tumor cells were confirmed by high MIB-1 expression (**Figure 3D**). Immunohistochemical staining also showed infiltration of CD8, but not CD4, positive lymphocytes into metastatic nodule (**Figures 3E–H**). The number of CD8 positive lymphocytes was increased in the metastatic tumor as compared with non-tumor area and that of CD4 (**Figure 3I**).

**Figure 4:**
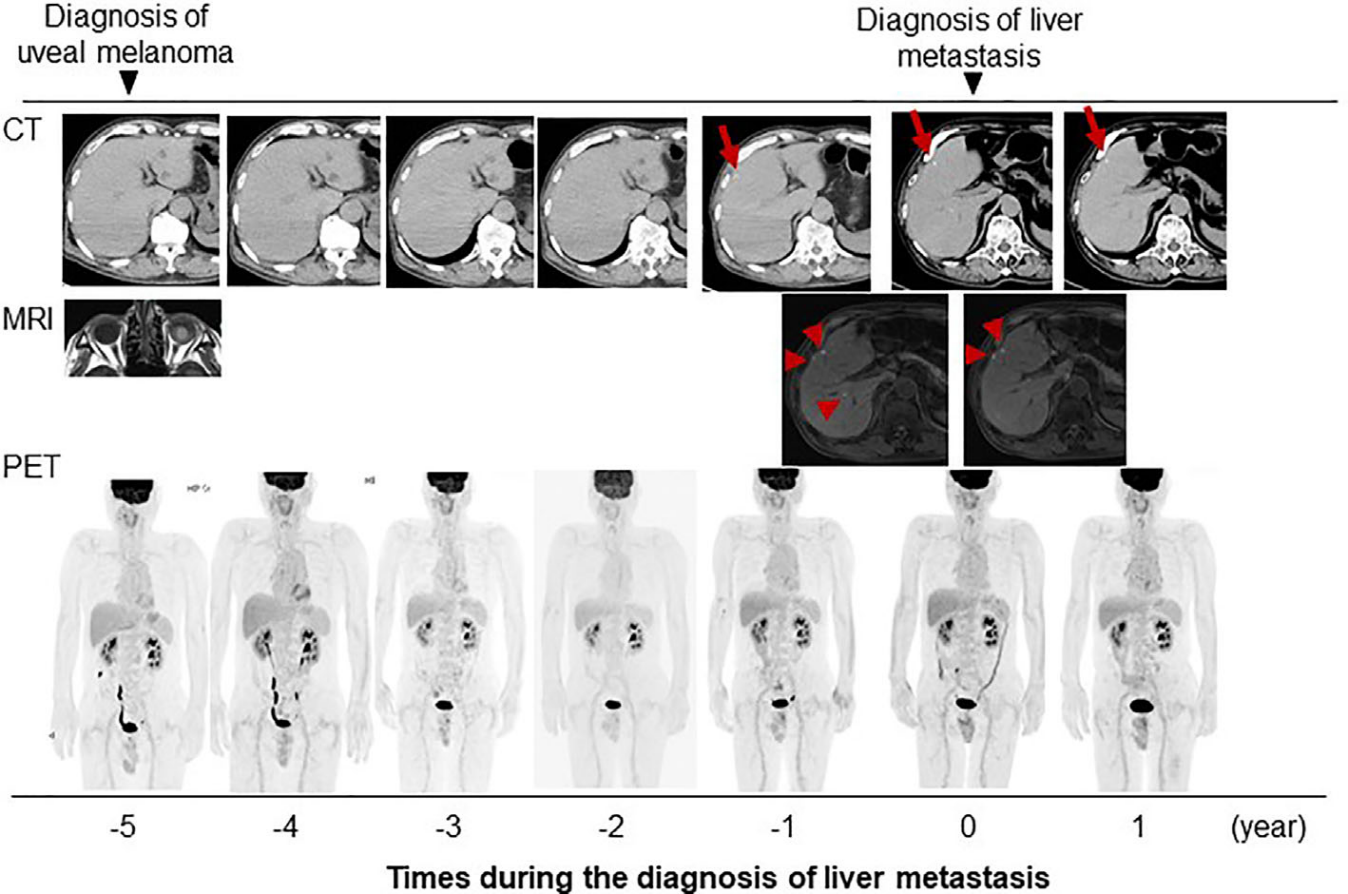
Clinical course of present case with images. Red arrow and arrow head indicate the metastatic tumors detectable with imaging studies.

Systemic chemotherapy with immune checkpoint inhibitors was proposed to the patient, but both he and his family refused aggressive treatment. The patient has been followed-up by CT and MRI at intervals of several months. He has experienced no change in the size or radiological pattern of these nodules for 1 year after presentation (**Figure 4**). He remains asymptomatic and remains in good condition without any treatment.

## Discussion

The present study describes a patient diagnosed with confirmed liver metastases of uveal melanoma. Despite receiving no treatment, this patient has remained asymptomatic, with no change in the size of the metastatic nodules for at least 1 year after presentation. Although the prognosis of patients with metastases of uveal melanoma is usually limited, adequate host immune response by CD8 positive lymphocytes might suppress disease progression.

FDG-PET/CT was inadequate in the early diagnosis of liver metastases in the present patient. Liver metastases of uveal melanoma were reported to be less sensitive to detection by FDG-PET/CT than liver metastases of cutaneous melanoma (13). Furthermore, FDG-PET/CT is relatively insensitive in the detection of small liver metastases (14), and the metastatic nodules in this patient were quite small, being only a few millimeters in diameter. Liver metastases of uveal melanoma have been reported to be hypodense on CT examination and to be enhanced with contrast, but these features are not specific to melanoma. Furthermore, small metastases are difficult to detect on CT examination (15).

Liver metastases often show high intensity on T1-weighted and diffusion-weighted MRI, various enhancement patterns on enhanced MRI and hypo-intensity on hepatobiliary phase (16–19). High-intensity on T1-weighted images of metastatic melanoma is thought to reflect intratumoral hemorrhage or certain sized areas of melanin pigmentation (16). However, only melanin pigmentation could induce high T1 intensity in the small metastases observed in the present patient, weakening its ability to diagnose metastatic lesions in the hepatobiliary phase of EOB-MRI. Liver metastases are hypo- or iso-echoic on B mode US, whereas contrast enhanced US shows strong enhancement in early arterial phase and is hypoechoic in delayed phase (14, 20). US and Sonazoid-enhanced US were effective for the detection and diagnosis of such small metastases in the present patient. Taken together, these findings indicate that MRI and contrast-enhanced US are useful for the detection and diagnosis of small liver metastases of malignant melanoma.

Effective systemic treatments for metastatic uveal melanoma have not been determined to date (20). Immunotherapy for uveal melanoma has been reported to be insufficient due to immune tolerance and low mutational burden (21, 22). The expression of programmed cell death ligand-1 is known to be associated with the infiltration of lymphocytes to the tumor and patient outcome (23). Immunosuppressive regulatory T cells and myeloid-derived suppressor cells reduce cell-mediated immunity in patients with liver metastases of uveal melanoma (22). Moreover, liver metastases induce resistance to immunotherapy through the depletion of CD8 positive T lymphocytes *via* macrophages (24). CD8 positive T lymphocytes in patients with advanced metastatic uveal melanoma have been detected only in peri-tumoral lesions, not but within tumors (25), reducing the efficacy of systemic treatment with immune checkpoint inhibitors. The liver metastases in the present case remained stable for 1 year after presentation, or 2 years after first detection on follow-up CT. Pathologic examination showed the infiltration of CD8 positive lymphocytes into these metastatic lesions.

Correlations between tumor infiltration by CD8 positive T lymphocytes and patient prognosis have been reported in patients with other types of malignant tumors (26, 27). For example, tumor infiltration by CD8 positive T lymphocytes was found to be significantly associated with disease-free survival and overall survival in breast cancer patients (26). CD8 positive T lymphocytes are thought to suppress micro-metastases in patients with colon cancer (27). Furthermore, intra-tumoral infiltration by CD8-positive, but not CD4-positive, lymphocyte has been associated with the prognosis and response to treatment in patients with malignant melanoma (28). The clinical course and pathology of the present patient suggest that the host immune response, including infiltration by CD8 lymphocytes, could suppress the progression of small-sized tumors. It is unclear when and why this immune suppression of metastatic tumors is disabled and these tumors progress. Immunotherapy may have shown a favorable anti-tumor effect in the present patient. Further careful observation of the present patient including molecular analysis of infiltrating CD8 lymphocytes, analysis of other immune cells such as NK cells or tumor microenvironment may provide clues to the optimal treatment of hepatic metastases of uveal melanoma.

In conclusion, this study described a patient with small hepatic metastases of uveal melanoma detected 5 years after primary tumor resection. MRI and contrast enhanced US could be useful in the early detection of hepatic metastases. This study also found that the hepatic metastases in the present patient were infiltrated by CD8 positive lymphocytes, and that, despite the absence of treatment, these metastases did not progress for 2 years. Although hepatic metastases of uveal melanoma have shown insufficient response to immunotherapy, host immunity may be sufficient.

## Data Availability Statement

The original contributions presented in the study are included in the article/supplementary material. Further inquiries can be directed to the corresponding author.

## Ethics Statement

Ethical review and approval was not required for the study on human participants in accordance with the local legislation and institutional requirements. The patients/participants provided their written informed consent to participate in this study.

## Author Contributions

KT designed the report. NT and KT wrote the manuscript. KT and KK collected the patient’s clinical data. KT was responsible for the conception and revision of the manuscript. KT and KK carried out the clinical management of the patient. ST performed pathological evaluation. IY organized this work. All authors contributed to the article and approved the submitted version.

## Conflict of Interest

The authors declare that the research was conducted in the absence of any commercial or financial relationships that could be construed as a potential conflict of interest.
